# Role of plasma fatty acid in age-related macular degeneration: insights from a mendelian randomization analysis

**DOI:** 10.1186/s12944-024-02197-8

**Published:** 2024-06-29

**Authors:** Guoge Han, Pinghui Wei, Meiqin He, Lanbo Jia, Qi Su, Xiru Yang, Rui Hao

**Affiliations:** 1 Clinical College of Ophthalmology, Tianjin Medical University, Tianjin, PR China; 2 Tianjin Key Lab of Ophthalmology and Visual Science, Tianjin Eye Hospital, Nankai University, Tianjin, PR China; 3 Nankai University Eye Institute, Nankai University Affiliated Eye Hospital, Nankai University, Tianjin, China; 4 First Teaching Hospital of Tianjin University of Traditional Chinese Medicine, National Clinical Research Center for Chinese Medicine Acupuncture and Moxibustion, Tianjin, PR China

**Keywords:** Mendelian randomization, Fatty acids, Age-related macular degeneration

## Abstract

**Background:**

An imbalance in lipid metabolism has been linked to the development of AMD, but the causal relationship between AMD and plasma fatty acids (FAs) remains controversial. Using a two-sample Mendelian randomization (MR) approach, we sought to evaluate the impact of specific FA plasma levels on the risk of different AMD subtypes.

**Methods:**

We analysed genome-wide association data of circulating FAs from 115,006 European-descended individuals in the UK Biobank. These data were used in a two-sample MR framework to assess the potential role of circulating FAs in developing wet and dry AMD. Sensitivity analyses were conducted to ensure the robustness of our findings. Additional multivariable and locus-specific MR analyses were conducted to evaluate direct effects of FA on AMD subtypes, minimizing biases from lipoprotein-related traits and triglycerides.

**Results:**

Mendelian randomization revealed associations of omega-3 was associated with decreased wet (OR 0.78, 95%CI 0.66–0.92) and dry AMD (0.85, 0.74–0.97) risk, showed a protective effect on AMD. Notably, the omega-6 to omega-3 ratio showed potential causal effects on both wet (1.27, 1.03–1.56) and dry AMD (1.18, 1.02–1.37). Multivariable MR suggested that the causal relationship of omega-3, omega-6 to omega-3 ratio on wet AMD persists after conditioning on HDL, LDL and triglycerides, albeit with slightly diminished evidence strength. Locus-specific MR linked to omega-3(*FADS1*, 0.89, 0.82–0.98; *FADS2*, 0.88, 0.81–0.96) and omega-6 to omega-3 ratio (*FADS1*, 1.10, 1.02–1.20; *FADS2*, 1.11, 1.03–1.20) suggests causal effects of these factors on wet AMD.

**Conclusions:**

The associations between plasma FA concentrations and AMD, suggest potential causal role of omega-3, and the omega-6 to omega-3 ratio in wet AMD. These results underscore the impact of an imbalanced circulating omega-3 and omega-6 FA ratio on AMD pathophysiology from MR perspective.

**Supplementary Information:**

The online version contains supplementary material available at 10.1186/s12944-024-02197-8.

## Introduction

Age-related macular degeneration (AMD) is a leading cause of visual impairment in the elderly population globally [1]. It is estimated that the number of AMD cases worldwide will rise to 288 million by 2040 [2]. While genetics contribute to AMD, environmental factors, especially dietary habits, also play a significant role [3]. AMD is categorized as either dry or wet, distinguished the presence or absence of choroidal neovascularization. Treatment for wet AMD typically involves repeated anti-VEGF drug injections, whereas treatment options for dry AMD, which aim to slow or reverse vision loss, are more limited [4, 5]. This highlights the need for preventive strategies for both forms of AMD.

Lipid accumulation, resulting from abnormal lipid metabolism, is recognised as a critical factor in AMD progression [6]. A key feature of dry AMD is the presence of lipid-rich deposits, known as drusen, situated between the retinal pigment epithelium and Bruch’s membrane, often described as a ‘hallmark’ of the disease [7]. Additionally, studies have shown a significant association between high levels of high-density lipoprotein cholesterol (HDL-C) and an increased AMD risk [8]. 

The impact of dietary lipids on AMD development has attracted research interest due to the potential role of oxidised or modified lipoproteins in drusen formation [9]. Epidemiological studies suggest that diets rich in n-3 polyunsaturated fatty acids (PUFA) and fish may lower AMD risk [10–12]. However, omega-3 fatty acid (FA) supplementation alone did not show benefits in the prevention wet AMD in the AREDS2 study [13, 14]. Cochrane systematic reviews of randomized controlled trials (RCTs) examining dietary advice or supplementation have raised questions about the hypnotized inverse relationship between omega-3 and AMD progression [15]. Furthermore, the influence of other fatty acids, such as monounsaturated fatty acids (MUFA) and saturated fatty acids (SFA), on AMD development is still unclear due to inconsistent and limited data [6, 16, 17]. While it is clear that abnormal lipid metabolism is linked to AMD pathogenesis, the underlying mechanism and causal relationships remain elusive. Further research is required to fully understand the role of lipids in the development and progression of AMD.

Mendelian randomization utilizes genetic variants as instrumental variables to determine the causal relationships between biomarkers and disease development. This method addresses some limitations inherent in traditional RCTs, including issues related to statistical power and bias. [18, 19] In contrast to previous studies that relied on self-reported dietary questionnaires, [17, 20] we obtained FA intake data from a large cohort of more than 115,006 participants from the UK Biobank. We therefore aimed to assess the association between plasma concentrations of various FA, including omega-3, omega-6, omega-6 to omega-3 ratio, linoleic acid (LA), docosahexaenoic acid (DHA), SFA, MUFA and PUFA, and the risk of developing two subtypes of AMD.

## Materials and methods

### Study design

In this study, we conducted a two-sample Mendelian randomization (MR) analysis to investigate the causal association between eight FA traits (omega-3, omega-6, omega-6 /omega-3 ratio; LA, DHA, SFA, MUFA, and PUFA) and two AMD phenotypes (wet and dry AMD). Dry AMD refers to non-exudative or atrophic form and wet AMD is characterized by macular exudative or neovascular form. Detailed information on the genome-wide association study (GWAS) summary data is provided in Table S1. The study adhered to the principles outlined in the Declaration of Helsinki. The original studies obtained ethical approval and informed consent and therefore no additional approval was required [21]. 

### Genetic instruments of fatty acid

The exposure GWAS data utilized in this study were obtained from the Integrative Epidemiology Unit (IEU) OpenGWAS project, available at https://gwas.mrcieu.ac.uk/. The IEU OpenGWAS project provides public access to genetic data from extensive GWAS. Notably, the GWAS data for FA traits included 115,006 participants of European cohort from the UK Biobank. [21]

Furthermore, to strengthen the validity of our results, we have incorporated data from alternative exposure sources to validate the impact of FAs (Omega-3 and omega-6 to omega-3 ratio) on AMD. We verified significant FA traits identified in the discovery stage. We chose GWAS ID: met-d-Omega_3 and met-d-Omega_6_by_Omega_3 as the replication exposure, which recruited 114,999 European samples.Criteria for selecting genetic instruments for each GWAS dataset were as follows: GWAS P-values < 5 × 10^− 8^; pairwise linkage disequilibrium (LD) between single nucleotide polymorphisms (SNPs) below an r^2^ threshold of 0.001 within a 10,000 kb clumping window. The F-value for instrumental variables was also calculated to include only instrumental variables with an F-value > 10, thereby reducing bias from weak instrumental variables.

### AMD outcome data

AMD outcome data were also obtained from the IEU OpenGWAS project. Data were extracted for two AMD subtypes, including dry AMD (GWAS ID: finn-b-DRY_AMD) and wet AMD (GWAS ID: finn-b-WET_AMD). The dry AMD GWAS dataset included 2,469 cases and 206,221 controls, whereas that for wet AMD comprised 2,114 cases and 206,601 controls. The IEU OpenGWAS project compiles data from various sources, such as population-based studies, offering a comprehensive dataset for research purposes. The studies included in the IEU OpenGWAS project vary in terms of sample size, geographic location and study design, thereby enhancing the generalizability of our findings.

### Statistical analysis

**Fig. 1:**
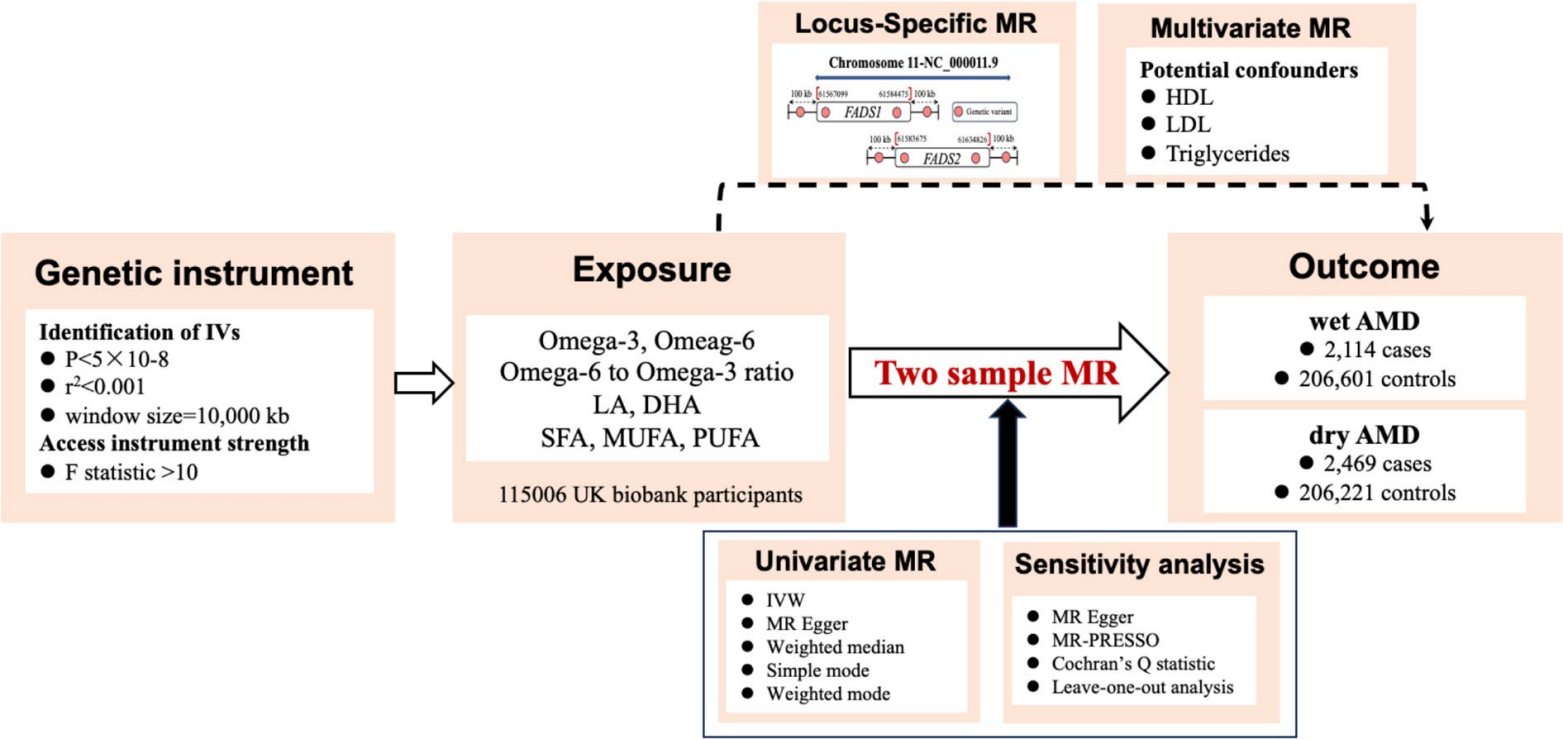
Flowchart of the Mendelian Randomization (MR) study

To identify potential causal relationship between the FA traits and AMD phenotypes, we performed two-sample MR analyses using the TwoSampleMR package (version 0.5.7) on the R statistical software (version 4.3.1). A multivariable MR design was also employed to assess the direct effects of FAs on AMD, controlling for potential confounders and mediators, including high-density lipoprotein (HDL), low-density lipoprotein (LDL), and triglycerides. Bonferroni correction was used to control the false discovery rate. For regional analyses, a Bonferroni-corrected P-value < 3.125 × 10^− 3^ [0.05/16] on both sides is regarded to indicate significance, a P value < 0.05 on both sides is regarded to indicate nominal significance, and a P-value between 3.125 × 10^− 3^ and 0.05 is considered as suggestive evidence. For a detailed overview and a visual representation of the process, refer to the flowchart in Fig. 1.

### Univariable mendelian randomisation analyses and sensitivity analyses

Following the harmonization of effect alleles and the exclusion of palindrome sequences, MR analyses were conducted. Outlier tests were used to detect and eliminate any outliers and ensure the reliability of the MR analysis results. The primary analyses utilized the inverse variance weighted (IVW) method to estimate causal effects, with a significance threshold set at *P* < 0.05. Odds ratios (ORs) and their 95% confidence intervals (CIs) were calculated to determine the direction (either positive or negative) of the causal effect. In the IVW method, the regression model intercept is assumed to be zero. Sensitivity analyses were performed to mitigate potential biases arising from directional pleiotropy. This was achieved by employing the MR-Egger method, which includes an intercept term (*P* < 0.05). In addition, the MR-PRESSO method was also used for outlier detection and to assess the overall heterogeneity of the MR estimates. Cochran’s Q test was employed to evaluate the heterogeneity across SNPs. In cases of heterogeneity, the results from the IVW method were examined using a multiplicative random-effects model. Furthermore, a leave-one-out analysis was conducted to evaluate the effect of individual SNPs on the overall MR findings.This process involved systematically excluding individual genetic variants one at a time and recalculating the MR-IVW estimates after each exclusion .

### *FADS1* and *FADS2* locus-specific MR

To further validate the association between FA traits and AMD, we employed an additional instrument using expression quantitative trait loci (eQTLs). SNPs within specific regions of the *FADS1* locus (chromosome 11: 61,567,099 to 61,584,475) and *FADS2* locus (chromosome 11: 61,583,675 to 61,634,826) were selected based on their significant association with FA levels (MAF > 1%, r^2^ < 0.30, *p* < 5.0 × 10^− 8^). A two-sample MR analysis using these eQTLs as instrumental variables was conducted to assess the causal relationship. This analysis aimed to determine whether the association between FA traits and AMD persisted when eQTLs were used as the instrument.

### Multivariable mendelian randomization

Furthermore, multivariable MR analysis was performed to account for the potential confounding effects from other lipid-related traits such as HDL, LDL and triglycerides. Genetic data for HDL (GWAS ID: ieu-b-109), LDL (GWAS ID: ieu-b-110), and triglycerides (GWAS ID: ieu-b-111) were sourced from the UK Biobank, which comprised a large European cohort of 403,943 to 441,016 individuals [22]. The multivariable MR analysis employed the IVW method. The potential confounders (e.g., HDL, LDL, and triglycerides) and adjustments were made for all exposures simultaneously in the model. A P-value < 0.05 was considered indicative of a significant causal relationship, suggesting a potential causal link between the FA traits and AMD, independent of HDL, LDL and triglycerides.

### Role of the funding source

The funding sources had no role in the design of the study, the collection, analysis, and interpretation of the data, the writing of the report, or the decision to submit the paper for publication.

## Results

### Instruments selection

For each set of fatty acid instruments, we harmonized the SNP-fatty acid and SNP-AMD data. Following the application of various quality control measures detailed in the Methods section, 30–65 SNPs were retained as instrumental variables for analysis. Each of these SNPs demonstrated acceptable validity with F-statistic values > 10. See Supplementary Table S2-S17 for more information on the remaining SNPs.

### Causal relationship between FFA and AMD

**Fig. 2:**
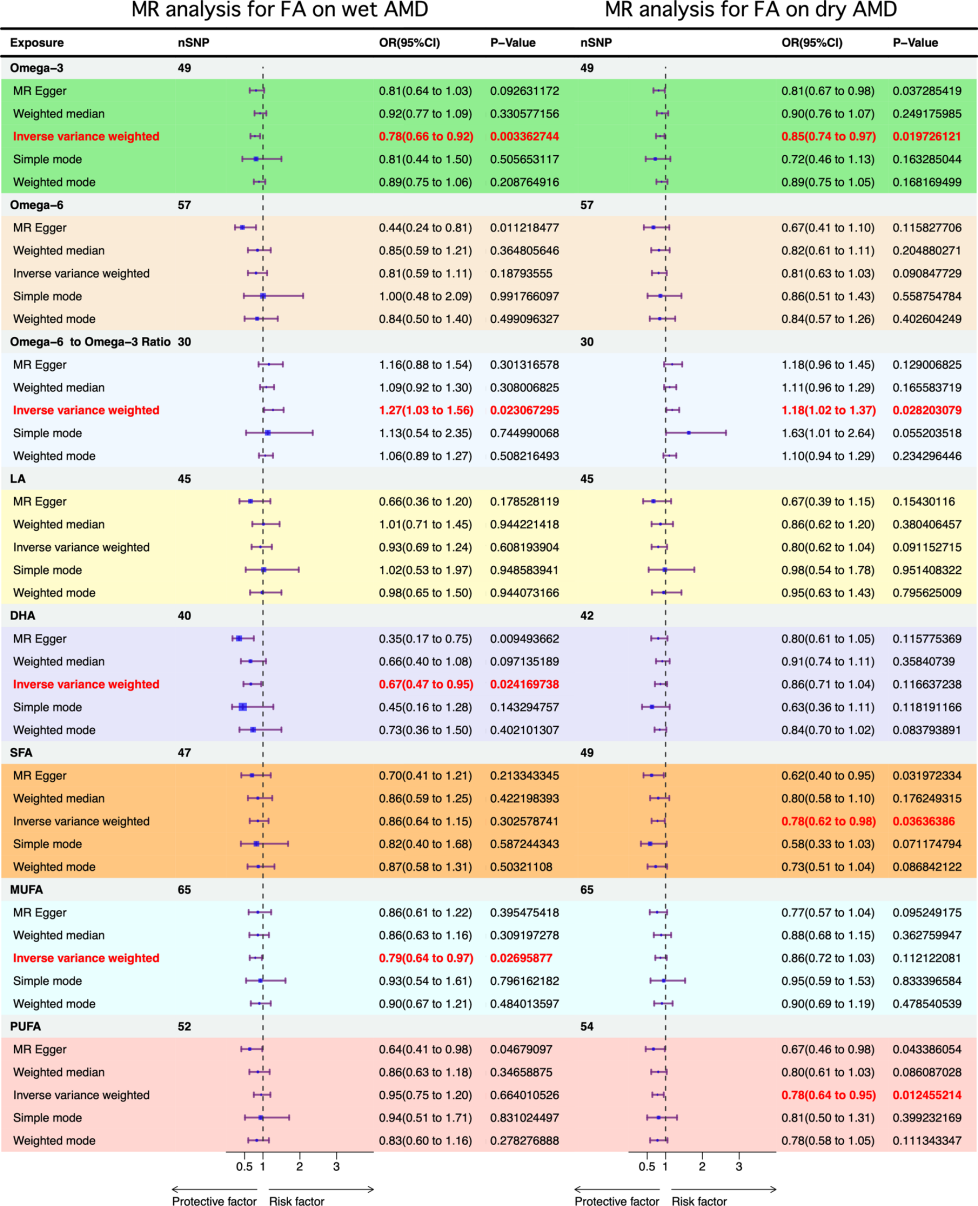
MR results of fatty acid with a causal relationship to AMD. In the graphs, significance rows (*P* < 0.05) are denoted in red. With OR = 1 as the reference line, the left side indicates that this FA is a protective factor for AMD, while the right side indicates that this FA is a risk factor for AMD. Abbreviations: MR: Mendelian randomization, AMD: age-related macular degeneration, SNP: single nucleotide polymorphism, OR: odds ratio, CI: confidence interval, LA: linoleic acid, DHA: docosahexaenoic acid, SFA: saturated fatty acid, MUFA: monounsaturated fatty acid levels, PUFA: polyunsaturated fatty acid levels

Univariate MR analysis revealed that a one standard deviation (SD) increase in omega-3 FA levels was associated with a decreased risk of both wet AMD (MR-IVW OR 0.78, 95%CI 0.66–0.92, P-value 3.4 × 10^− 3^) and dry AMD (MR-IVW OR 0.85, 95%CI 0.74–0.97, P-value 1.97 × 10^− 2^) (Fig. 2). Interestingly, no causal relationship was observed between omega-6 fatty acids and wet or dry AMD. However, a higher omega-6 to omega-3 FA ratio was linked to an increased risk of both wet AMD (OR 1.27, 95%CI 1.03–1.56, P-value 2.31 × 10^− 2^) and dry AMD (OR 1.18, 95%CI 1.07–1.37, P-value 2.82 × 10^− 2^).

In contrast, we observed different responses to SFA and MUFA levels in the two types of AMD. Elevated SFA levels were associated with a reduced risk of dry AMD (OR 0.78, 95%CI 0.62–0.98, P-value 3.64 × 10^− 2^), but showed no causal link with wet AMD. In contrast, increased monounsaturated fatty acid levels lowered the risk of wet AMD (OR 0.79, 95% CI 0.64–0.97, P-value 2.70 × 10^− 2^) without exhibiting a causal relationship with dry AMD.

Additionally, higher DHA correlated with a decreased risk of wet AMD (OR 0.67, 95%CI 0.47–0.95, P-value 2.41 × 10^− 2^), but had no impact on dry AMD. Increased PUFA were linked to a reduced risk of dry AMD (OR 0.78, 95% CI 0.64–0.95, P-value 1.24 × 10^− 2^). No causal relationship was found between LA levels and either wet or dry AMD.

### MR sensitivity analysis

The MR sensitivity analysis are detailed in Table S18. The MR-Egger intercept suggested the presence of horizontal pleiotropy in the analysis of the causal relationship between PUFA and wet AMD. However, no evidence of horizontal pleiotropy was found in the other analyses. Mild to medium heterogeneity was observed in certain outcomes, such as omega-3, omega-6, LA, and SFA, as indicated by Cochran’s Q test.

Scatter and funnel plots, shown inSupplement Figure S3, demonstrate the consistent causal effects observed in our study. Forest plots present individual SNP outcome estimates, and leave-one-out analysis, depicted in Supplementary Figures S1–S2, reveals the impact on outcome when each SNP is excluded.

**Fig. 3:**
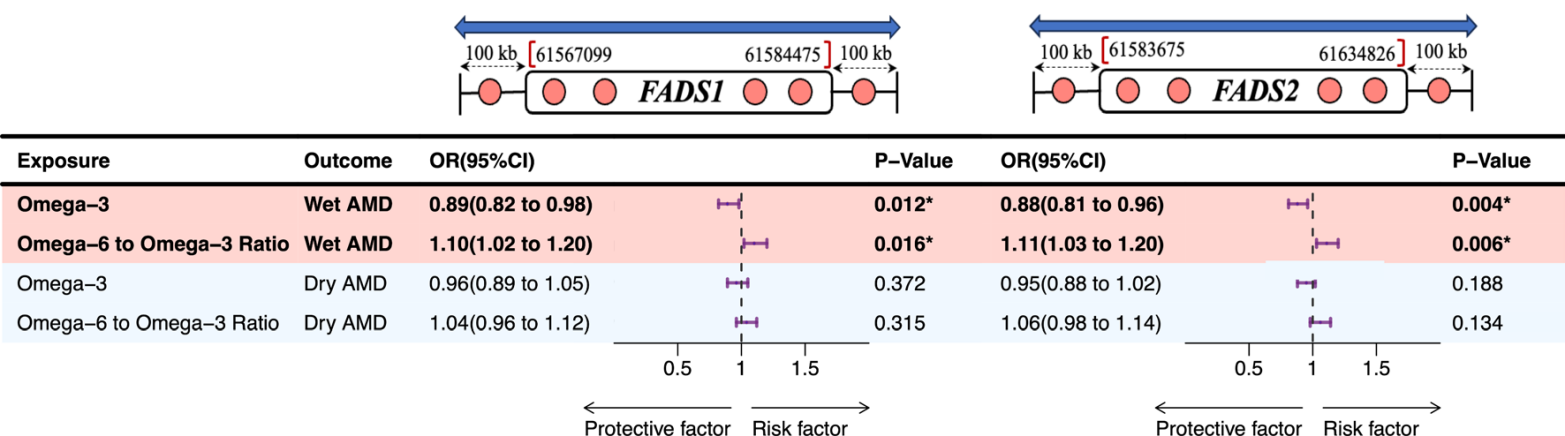
Causal effects of fatty acids on AMD through the influence of the *FADS1* and *FADS2* gene cluster. The odds ratios of AMD are presented based on a standard deviation increases in serum omega-3 and omega-6 to omega-3 ratio measures. These estimates were obtained using SNP instruments within the *FADS1* and *FADS2* gene cluster. SNP: single nucleotide polymorphism

Subsequently, an analysis of SNPs within the *FADS1* and *FADS2* clusters to evaluate their impact on AMD. The results of *FADS1* and *FADS2* SNPs analyses also demonstrated that omega-3 concentrations were associated with a decreased risk of wet AMD, as depicted in Fig. 3. In contrast, the omega-6 to omega-3 ratio was associated with an elevated risk of wet AMD, as shown in Fig. 3.

### Causal association based on MR results in the replication stage

The results indicate that omega-3 (OR 0.77, 95% CI 0.66–0.92, P-value 3.20 × 10^− 3^) has a protective effect on wet AMD and an increase in omega-6 to omega-3 ratio (OR 1.23, 95% CI 1.02–1.49, P-value 3.34 × 10^− 2^) poses a risk of promoting the progression of wet AMD. For dry AMD, only omega-3 (OR 0.84, 95% CI 0.74–0.95, P-value 7.48 × 10^− 3^) showed a protective effect and the effect of omega-6 to omega-3 ratio in promoting disease progression disappeared. This further confirms the reliability of our previous results. No heterogeneity and pleiotropy were detected in the replication stage.

### Multivariable mendelian randomization

**Fig. 4:**
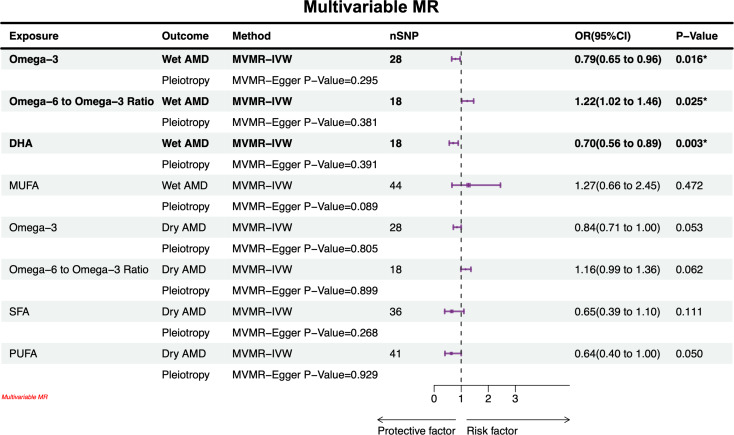
Results of multivariable MR analysis of balanced confounders HDL, LDL, and triglyceride. Attention: In the graphs and tables, significance is denoted as follows: * for < 0.05

In the multivariable Mendelian randomization analyses, the IVW method was used to validate the observed causal effect of FA concentration on AMD conditioned on HDL, LDLand triglyceride levels. After adjusting for these confounding factors, omega-3 (OR 0.79, 95% CI 0.65–0.96) and DHA (OR 0.70, 95% CI 0.56–0.89) continued to exhibit protective effects in individuals with wet AMD. However, a higher omega-6 to omega-3 ratio was associated with an increased risk of wet AMD (OR 1.22, 95% CI 1.02–1.46). However, MUFAs no longer demonstrated a protective role. Notably, no causal relationship was found between any type of FA and dry AMD, as depicted in Fig. 4.

## Discussion

In this study, we employed two-sample MR to explore the potential causal relationship between plasma FA concentrations and AMD risk. The results indicate that a higher omega-6 to omega-3 ratio was associated with an increased AMD risk. Conversely, higher plasma concentrations of omega-3 fatty acids were associated with a lower risk for both AMD types. However, the protective effects of omega-3 were somewhat attenuated for dry AMD after adjusting for other lipids (e.g. HDL, LDL, and triglycerides) using Multivariable Mendelian randomization. Subsequent *FADS* locus-specific MR analyses suggested that these relationships are likely driven by genetic variants within the *FADS* gene cluster, which plays a role in PUFA desaturation.

Omega-3, derived from alpha-linolenic acid, includes essential fatty acids like DHA, which cannot be synthesised in humans. [23] These acids perform essential structural and protective functions in the retina. [6, 24] For example, DHA, highly concentrated in photoreceptors’ outer segment membranes, is crucial for cell survival and maintaining retinal homeostasis [25, 26]. Additionally, DHA derivatives, such as neuroprotectins, have anti-inflammatory and anti-angiogenic properties [27–29], crucial in AMD where local antioxidant capacity is vital in the macular area [30]. 

Previous research, encompassing cross-sectional, cohort studiesand meta-analyses, suggests that high omega-3 intake may reduce the risk of AMD [6]. However, systematic reviews by Cochrane, including RCTs, concluded that increasing dietary omega-3 levels does not significantly prevent AMD progression [31, 32]. Subsequent RCTs, such as tAREDS2 and NAT2, investigating long-chain omega-3 oral supplementation, have produced conflicting results, influenced by factors like baseline nutritional parameters, supplementation formula, and bioavailability. [14, 33] Interestingly, wet AMD progression was prevented in NAT2 participants who maintained elevated plasma and cellular DHA levels [34, 35]. Similarly, the current MR study provides evidence that higher circulating plasma concentrations of omega-3, particularly DHA, may lower the risk of wet AMD.

Several factors must be considered to explain the inconsistencies among these findings. First, the result of the MR study regarding the effects of DHA or omega-3 fatty acids on AMD should be interpreted in the context of long-term or lifetime exposure [19]. For example, *FADS* genotypes affect children’s DHA supply during pregnancy via maternal RBC phospholipids [36]. In contrast, the impact of short-term oral PUFA supplementation in RCTs or dietary intake studies (typically 3–5 years) may not provide sufficient protective effects against AMD. This outcome may also partly depend on the overall diet composition or individual dietary habits of the participants. Second, dietary intake data of PUFA composition does not fully reflect plasma concentrations or lipid concentrations in photoreceptors’ outer segments [6]. Other mechanisms, such as phospholipase A2 activity, oxidative stress, and local trafficking, might contribute to the contradictory data beyond mere synthesis or intake [27, 37–39]. Third, the omega-3 supplementation formula and administration time are essential for treatment outcome [40, 41]. Future studies, designed with a focus on causal inference, are required to optimize specific FA concentrations/formulas at different illness stages and their effects in response to treatment. The current MR results at least provide valuable insights into which supplements might be effective against AMD.

Compared with omega-3, a relatively high proportion of dietary omega-6 consumption has detrimental effects on AMD, which is supported by heterogeneous and inconsistent evidence. A few epidemiological studies have indicated that a high intake of omega-6 is significantly associated with the risk of AMD. [6, 42, 43] In contrast, an elevated plasma omega-6 to omega-3 ratio, leading to an imbalance in circulating oxidation products, was correlated with AMD risk in a Chinese cohort [27]. These contradictory results might arise because the ratio calculated from dietary intake data cannot be directly equated with values determined from plasma. Our MR analysis suggests that the omega-6 to omega-3 ratio may have a positive causal effect on wet AMD. Notably, our findings align with the NAT2 RCT cohort study, which found a higher omega-6 to omega-3 ratio in patients with AMD [34]. This supports the hypothesis that a disproportionate dietary omega-6 to omega-3 ratio (such as the 20:1–30:1 ratio common in Western diets) may contribute to AMD pathology, particularly by accelerating inflammation and oxidative stress.

In addition to individual dietary preferences for FA intake, genetic factors have been associated with the composition of circulating fatty acids, especially in plasma [44]. Genome-wide association studies have shown that variations in the FADS haplotype, which encodes the Δ-5 and Δ-6 desaturases, are linked to PUFA blood concentrations and disrupted long-chain PUFA synthesis in conditions such as cardiovascular diseases, schizophrenia and major depressive disorder [18, 19, 45]. *FADS* enzymes are directly linked to both omega-3 and omega-6 long-chain PUFA metabolism and play a central role in the ratio-limiting steps of their respective pro-inflammatory and anti-inflammatory precursor lipid metabolites. [46, 47] Our MR analysis also suggests possible causal effects of omega-3, DHA and the omega-6 to omega-3 ratio on wet AMD via the FADS gene cluster. Consequently, it is speculated that in regions with limited omega-3 availability or scarce access to seafood, *FADS* genetic variants may exacerbate the omega-6 to omega-3 ratio imbalance typical of modern Western diet preferences, ultimately leading the progressive onset of wet AMD.

Studies have also investigated the effect of these FA on dry AMD endpoints. Although previous animal or clinical RCT studies have supported this, [48, 49] the causality between omega-3 and dry AMD was attenuated after multivariant MR correction. This is partly because some FA instruments share loci also linked to lipoproteins, suggesting that we cannot propose a direct effect of omega-3 and the omega-6 to omega-3 ratio independent of HDL, LDL, and triglycerides on dry AMD.

Our study has several strengths. First, it employs the largest available GWAS samples for identifying FA instruments and evaluating causation, thereby minimizing bias from small sample sizes and traditional observational study methodologies. Second, the consistent effect power observed across heterogeneity, horizontal pleiotropy, and multivariable MR analyses reinforces the impact of omega-3 and DHA on wet AMD. Last, distinct MR approaches were utilized to validate the effects more robustly, reducing the potential influence of horizontal pleiotropy.

However, our data must be interpreted within the context of limitations. One notable limitation is the focus on individuals of European ancestry. It is important to acknowledge that the frequencies of *FADS* risk alleles differs across geographic regions [50, 51]. In fact, divergent *FADS* haplotypes are more commonly observed in populations of non-European descent, such as African Americans [44, 52]. Consequently, the mechanistic effects proposed in our study might be more pronounced in these non-European ancestral groups. Second, our study does not provide specific insights into the optimal dosage or formula ratio of polyunsaturated fatty acids, nor does it address the ideal timing for their supplementation. Therefore, future studies should investigate the omega-6 to omega-3 ratio range associated with wet AMD risk. Thirdly, a comprehensive MR analysis would ideally include a broader range of FA. In this study, typical and significant FA metabolites were quantified. However, quantification of other metabolites, such as arachidonic acid, was not feasible due to the limited number of SNPs available instrument screening.

In conclusion, our MR findings support the protective role of circulating DHA and omega-3 against the risk of wet AMD. Furthermore, this study suggests a role for an increased omega-6 to omega-3 ratio in the pathophysiology of AMD. However, the causal relationship was attenuated in dry AMD samples after multivariable MR conditioned on other lipids.

### Electronic supplementary material

Below is the link to the electronic supplementary material.

Supplementary Material 1

Supplementary Material 2

Supplementary Material 3

Supplementary Material 4

## Data Availability

The datasets generated or analysed during the current study are available in OpenGWAS database (https://gwas.mrcieu.ac.uk/).
