# Supplementary material for: Role of plasma fatty acid in age-related macular degeneration: insights from a mendelian randomization analysis
Source: Lipids Health Dis. 2024 Jun 29;23:206. doi: 10.1186/s12944-024-02197-8 (PMC11218068; doi:10.1186/s12944-024-02197-8)
Supplement: Supplementary file 2 — Supplementary Material 2 [file 12944_2024_2197_MOESM2_ESM.docx]

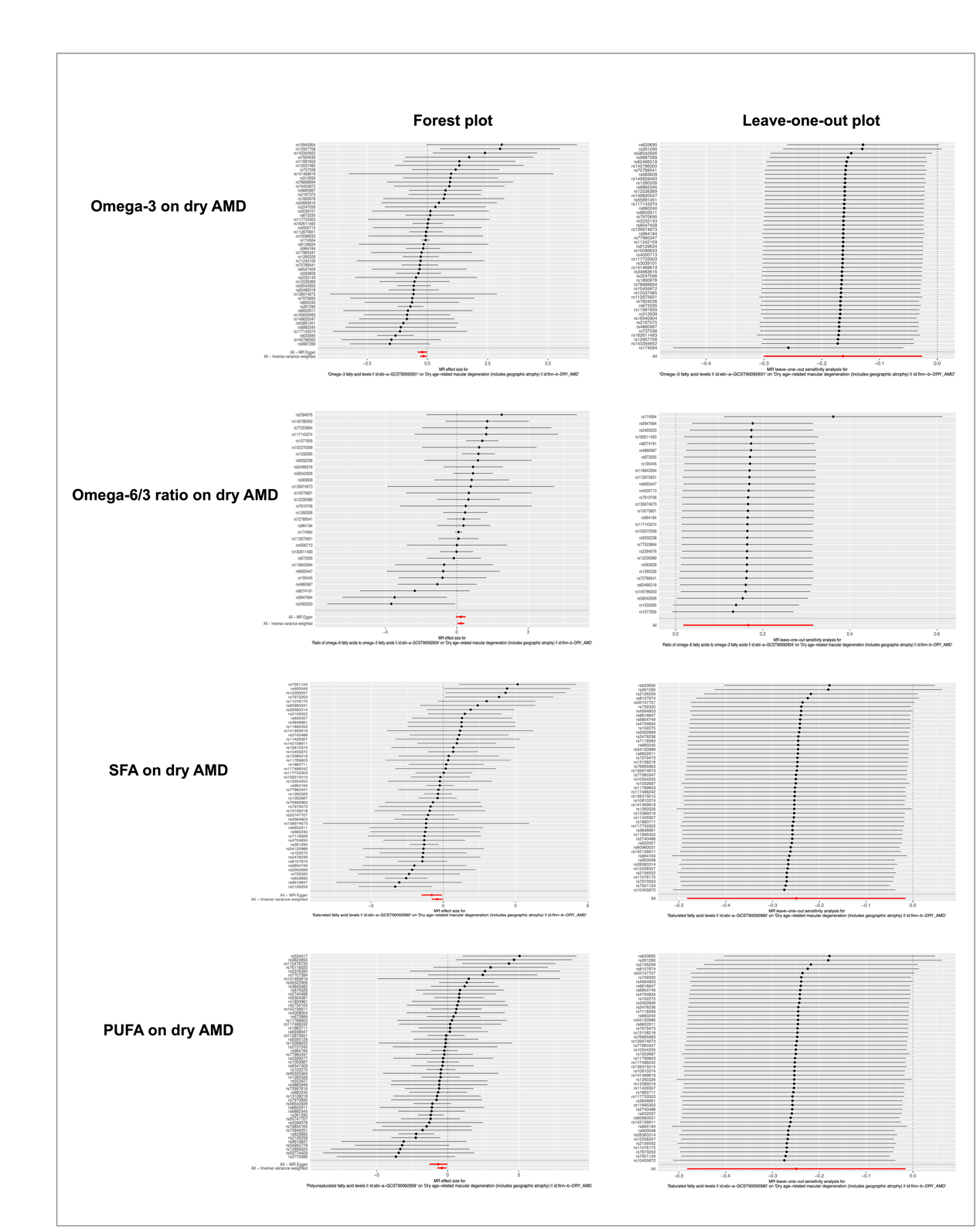


**Figure S2.** The forest plot and leave-one-out plot illustrating the predicted effects of fatty acid on dry AMD. In the leave-one-out plot, after removal of each SNP, one at a time, did not have a significant impact on the analysis results of the remaining SNPs.
