# Supplementary material for: Role of plasma fatty acid in age-related macular degeneration: insights from a mendelian randomization analysis
Source: Lipids Health Dis. 2024 Jun 29;23:206. doi: 10.1186/s12944-024-02197-8 (PMC11218068; doi:10.1186/s12944-024-02197-8)
Supplement: Supplementary file 4 — Supplementary Material 4 [file 12944_2024_2197_MOESM4_ESM.docx]

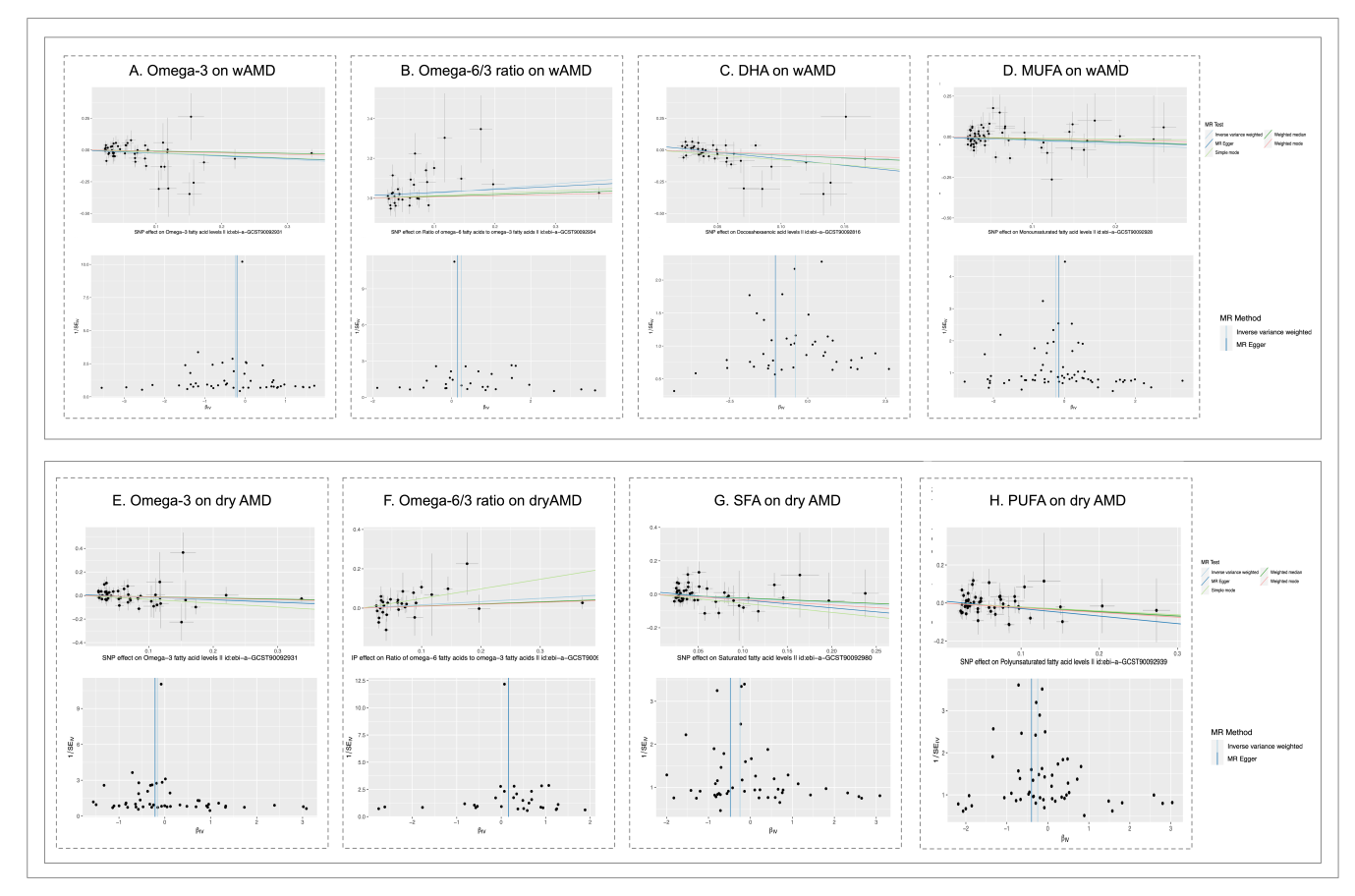


**Figure S3**. The scatter plot and funnel plots illustrating the predicted effects of fatty acid on AMD. Each line’s slope in the scatter plot represents the estimated MR effect per method. The funnel plot indicates that the causal relationship is unlikely to be affected
